# Influence of Social Media on Alcohol Use in Adolescents and Young Adults

**DOI:** 10.35946/arcr.v36.1.09

**Published:** 2014

**Authors:** Megan A. Moreno, Jennifer M. Whitehill

**Affiliations:** Megan A. Moreno, M.D., M.S.Ed., M.P.H., is an associate professor at the University of Washington and at the Seattle Children’s Research Institute, Center for Child Health Behavior and Development, Seattle, Washington. Jennifer M. Whitehill, Ph.D., is an assistant professor in the Department of Public Health, University of Massachusetts Amherst, Amherst, Massachusetts.

**Keywords:** Alcohol use, abuse, and dependence, underage drinking, risky drinking, portrayal of alcohol and other drug use (AODU) in the media, prevention, intervention, adolescent, young adult, technology, Internet, online social media, marketing, social marketing, message, Facebook, Twitter, Social Learning Theory, Media Practice Model, Facebook Influence Model

## Abstract

Participation in online social media Web sites (e.g., Facebook and Twitter) has skyrocketed in recent years and created a new environment in which adolescents and young adults may be exposed to and influenced by alcohol-related content. Thus, young people are exposed to and display pro-alcohol messages and images through online portrayals of drinking on personal pages as well as unregulated alcohol marketing on social media sites that may reach underage people. Such online displays of alcohol behavior have been correlated with offline alcohol behavior and risky drinking. Health behavior theories have been used to describe the influence of social media sites, including Social Learning Theory, the Media Practice Model, and a more recent conceptual approach called the Facebook Influence Model. Researchers are beginning to assess the potential of social media sites in identifying high-risk drinkers through online display patterns as well as delivering prevention messages and interventions. Future studies need to further expand existing observational work to better understand the role of social media in shaping alcohol-related behaviors and fully exploit the potential of these media for alcohol-related interventions.

Today’s generation of adolescents and young adults are growing up immersed in social media, such as Facebook and Twitter, that promote user-generated content and interactions between users (Lenhart et al. 2005). The use of such media is especially high among these age groups (Madden et al. 2013*b*). Social media sites are an environment in which alcohol-related content is frequently created and consumed by adolescents and young adults (Moreno et al. 2009*a*,b, 2010). Displayed alcohol references on social media may include information and images pertaining to alcohol (Hinduja and Patchin 2008; Moreno et al. 2009*b*, 2010*a*) that may influence viewers (Litt and Stock 2011; Moreno et al. 2009*a*) and be indicative of offline alcohol use (Moreno et al. 2011). This article discusses social media, their popularity, and their social nature that promotes information sharing and peer connections. It also reviews health behavior theories that support the influential nature of social media, including a newer conceptual approach called the Facebook Influence Model. Finally, the article describes first efforts to use social media for alcohol prevention and intervention and explores how future work could enhance such efforts through observational studies and intervention development. The discussion focuses largely on Facebook and Twitter, two of the most popular social-networking sites at present, because the greatest volume of research has been published about these sites. Throughout the discussion, the review emphasizes the characteristics that make social media social: their interactive nature, the presence of user-generated content, and the formation of networks.

## Social Media Sites

**Figure 1:**
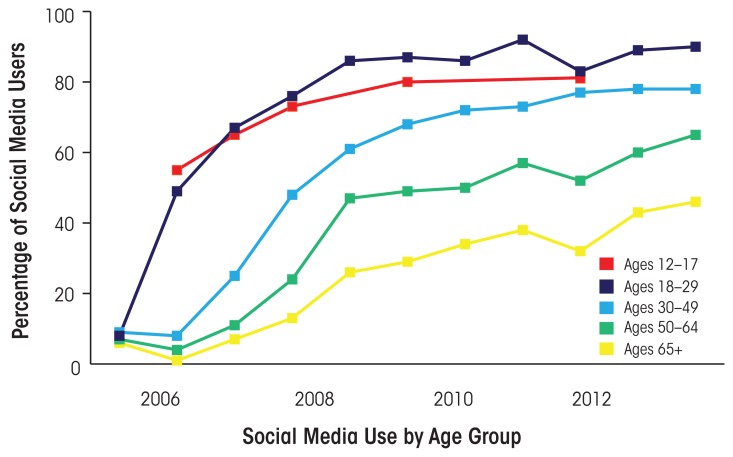
Changes in social media use among Internet users by age group. SOURCES: Madden, M.; Lenhart, A.; Cortesi, S.; et al. *Teens, Social Media, and Privacy.* Washington, DC: Pew Research Center, 2013*a.* Pew Research Center. *Data Trend: Social Media Use by Age Group Over Time*. Washington, DC: Pew Research Center, 2014. Available at: http://www.pewinternet.org/data-trend/social-media/social-media-use-by-age-group/ Accessed January 4, 2015.

Social media use has grown exponentially over the past decade, and this growth is expected to continue (see figure 1) (Duggan and Smith 2013). This section provides an overview of social media use and trends, with a focus on Facebook and Twitter.

### Social Media Are Interactive

Social media sites are diverse and yet share many similar features. Site users generally create an account; link to a network of other individuals or groups; and use the site to share thoughts, photographs, videos, news stories, and other content (Kietzmann et al. 2011). Social media can be used by individuals to share information about their personal lives as well as by businesses and organizations to promote their products and services. Most of the sites have built-in mechanisms to express approval or disapproval of content; consequently, users can not only form their own impression of a post or video but also can see how many others, and sometimes exactly who, also expressed approval. This multidirectional and user-generated communication about content differentiates social media from traditional mass media and from the earlier days of Internet advertising, when Web sites generally just provided content from one entity or posted information about a product (Kaplan and Haenlein 2010).

### The Changing Landscape of Social Media

To understand how alcohol-related messages and images displayed on social media may influence young people, it is important to consider the changing landscape of social media. Different social media sites have gained and lost popularity over time, and new ones are continually being launched to cater to specific market niches and demands, leading to a constantly changing landscape of sites and mobile applications. MySpace is one of the older social media sites, with an emphasis on music sharing; it was among the most popular sites globally in the early 2000s (Lenhart and Madden 2007). Facebook was launched in 2004; it initially was available only to students at Harvard University but quickly spread to other colleges and by 2006 was available to the general public. As Facebook expanded beyond its roots as a network only for college students, MySpace’s dominance began to decline. Then, in 2006, Twitter emerged with an emphasis on short text messages (Lenhart et al. 2010). In addition to these popular social-networking sites, sites focused on professional networks (e.g., LinkedIn), photo sharing (e.g., Instagram, Snapchat, Pinterest), video sharing (e.g., YouTube, Vimeo), and other niches have arisen.

### Facebook and Twitter: Popularity, Access, and Privacy

Facebook and Twitter are among the most-visited Web sites in the United States, particularly among adolescents and young adults. As of 2013, 77 percent of adolescents used Facebook and 24 percent used Twitter (Madden et al. 2013*b*); among young adults, the corresponding percentages were 86 percent and 27 percent (Duggan and Brenner 2013). As a result, any alcohol-related content posted on these sites has the potential to reach a large proportion of adolescents and young adults. Several characteristics of social media sites can influence this risk of exposure to alcohol content, including the formats available for user posts and the options for and culture of anonymity and privacy. These issues are especially salient given that references to personal drinking could be incriminating for individuals under age 21. This section compares Facebook and Twitter with respect to these domains.

Over 1 billion people worldwide use Facebook (The Nielsen Company 2013). The site specifies a minimum age of 13 to participate in the network and requires the user to enter his or her age when creating an account, but there is evidence that children under age 13 participate in Facebook by providing a false age (Jernigan and Rushman 2014; Richtel and Helft 2011). When establishing an account, the Facebook user can create a profile listing numerous aspects of his or her identity, including birthday, hometown, schools attended, jobs held, and relationship status, which indicates whether someone is in a romantic relationship. Facebook requests that each user register with his or her real name and then use that full name as the identifier for the profile. An overwhelming majority (94.9 percent) of college students use their real names on Facebook (Tufekci 2008). Use of real names helps users identify and connect with individuals whom they know offline.

The Facebook experience in 2014 centers on the user’s “wall” or “timeline,” where he or she displays status updates, photos, and other items. Users can control who is able to see the content on their timeline through a robust set of privacy settings. A majority of teens on Facebook report using these privacy settings (Madden et al. 2013*a*), but some studies suggest that adolescents may overestimate their understanding of how to establish and maintain private settings (Moreno et al. 2012*b*).

Twitter is less commonly used than Facebook, with 215 million active users in 2013 (Kim 2013). Twitter posts, or tweets, are text messages of no more than 140 characters. Although adolescent participation in Twitter currently is less than participation in Facebook, the number of young users of this site is rapidly growing (Madden et al. 2013*b*). Twitter content often includes a hashtag, connoted by the pound sign followed by a keyword (e.g., #party, #beer). Keywords serve a unique function because they can be searched within Twitter by users to find content related to a particular topic. In contrast to Facebook, Twitter does not ask for the user’s age when creating an account, although their policies state that accounts of users discovered to be under age 13 will be deactivated. Madden and colleagues (2013*a*) found that 36 percent of 12-year-old Internet users reported falsifying their age to access a Web site or account. Twitter’s privacy settings are limited to either making content fully public or sharing it only with “followers” of the account. Twitter executives have said that 90 percent of the content on the site is fully public (Rao 2010). In 2013, only 24 percent of teen Twitter users reported keeping their tweets private, whereas 60 percent kept their Facebook profiles private (Madden et al. 2013*b*). Part of teens’ willingness to disclose information publicly on Twitter may stem from the fact that the company does not make any requests to use a person’s real name as the online username.

Both Facebook and Twitter are being used for research purposes, but with somewhat different modes of analysis. Thus, Facebook often is considered as a platform in which the unit of analysis is an individual identity expressed via a profile. In contrast, Twitter frequently is considered to be a platform in which the unit of analysis is a specific topic around which individual users may interact, congregate, or “follow.”

## Alcohol Content on Social Media

For young people, social media are a source of exposure to two important factors that offline are associated with alcohol use: peer alcohol behavior (Ali and Dwyer 2010; Mundt et al. 2012) and alcohol advertising (Jernigan 2006, 2011). Alcohol researchers have begun to measure exposure to and impact of alcohol-related content and are moving toward developing intervention mechanisms using social media. However, the ways in which social media exposure may be similar to, different from, or reinforcing of offline counterparts are not yet fully understood.

### User-Generated Alcohol Content

Content posted by adolescents and young adults likely is seen by peers as well as younger users of these sites. Early studies on the effects of this exposure focused on MySpace; however, research efforts have kept pace with changes in the popular social media platforms to include Facebook and Twitter. Several studies have illustrated that adolescents’ displays on social media (i.e., MySpace and Facebook) frequently include portrayal of health-risk behaviors related to alcohol, other substances, and sexual behaviors (Hinduja and Patchin 2008; McGee and Begg 2008; Moreno et al. 2007, 2009*b*). Alcohol-related displays may include texts (e.g., “Matt got drunk last night”), photographs depicting alcohol consumption, or links to alcohol-related groups or companies (Egan and Moreno 2011; Moreno et al. 2010*a*).

The patterns of displaying such health-risk behaviors online commonly are consistent with offline reporting. For example, adolescents who display one health-risk behavior (e.g., sexual activity) on social media are more likely to also display other behaviors (e.g., alcohol use) (Moreno et al. 2009*a*). Also, risk behaviors may be displayed online within peer groups, just as offline peer groups commonly report engagement in similar behaviors. Thus, adolescents are more likely to display references to sexual behavior if a peer displayed similar references (Moreno et al. 2010*b*). Finally, displayed alcohol references have been linked to alcohol behaviors offline, because older adolescents whose Facebook posts suggested problem drinking behaviors are more likely to score as “at risk” on a problem-drinking screen (Moreno et al. 2011).

Whereas health-risk behaviors commonly are displayed on social media sites, negative consequences of these behaviors are not frequently noted. In a study of older adolescents, displays of negative consequences of alcohol use, such as hangovers or embarrassment, on social media sites were rare (Moreno et al. 2010*a*).

More recently, researchers have begun to examine alcohol-related content on Twitter, which provides a more immediate reflection of behaviors as they occur. The extent to which social networks are used in real time to discuss alcohol has implications for surveillance and intervention. Previous studies in other health-related areas have illustrated that Twitter can be used to identify behaviors or intentions across populations (Chew and Eysenbach 2010; Signorini et al. 2011). One study (West et al. 2012) examined keywords that are synonyms for the word “drunk” among a sample of over 5 million tweets from users selected to be geographically representative of the U.S. population. The investigators found that tweets related to intoxication peaked between the hours of 10 p.m. and 2 a.m. in the user’s local time zone and were more prevalent on Friday and Saturday nights. Moreover, the proportion of tweets related to intoxication was 0.53 percent over the New Year’s holiday weekend, compared with 0.34 percent during non-holiday weekends. These findings are consistent with studies emphasizing the increased risk for alcohol problems during holidays and other specific events (Neighbors et al. 2011). Thus, at the population level, the timing of tweets about alcohol behaviors correlates with the times when the heaviest drinking and highest proportion of alcohol-related motor vehicle crashes are known to occur. Additional research is needed to examine these findings with other alcohol-related keywords and behaviors and to test, at the individual level, whether tweets about intoxication and impaired driving are correlated with risky drinking behaviors.

### Unregulated Marketing on Social Media

In addition to user-generated alcohol-related content, there is growing concern about the extent to which adolescents and young adults are exposed to alcohol marketing on social media sites. Research from both the United States and the United Kingdom indicates that the major alcohol brands maintain a presence on Facebook, Twitter, and YouTube (Jernigan and Rushman 2014; Winpenny et al. 2014).

Analysis of social media marketing for leading alcohol brands in the United Kingdom has identified the most common marketing strategies, including promotion of offline branded events (e.g., at a club or sporting event), interactive games, sponsored online events, and invitations to drink (Nicholls 2012). On Facebook, alcohol companies ask users to “like” their brands and to post pictures of themselves drinking the specific alcohol beverage or participating in real-life events sponsored by the company. On Twitter, brands are encouraging followers who attended an event to post pictures of themselves using a dedicated hashtag, thereby enforcing the brand’s identity among Twitter users. This practice is of particular concern given the popularity of Twitter among younger teens. Other examples of advertising on Twitter included tweets noting that it is a specific day of the week on which is a good time to drink a specific brand of alcohol, such as the Bacardi brand using the hashtag #mojitomonday. In contrast, only two of the five brands analyzed included a small number of tweets encouraging followers to drink responsibly and get home safely (Nicholls 2012).

Although restrictions exist to protect young people from exposure to alcohol advertisements on traditional media channels (e.g., recommendations to limit alcohol commercials during youth-oriented television programming) (Ross et al. 2014), adolescents still have access to alcohol advertising in many traditional venues (King et al. 2009; Rhoades and Jernigan 2013). Social media present a new venue for alcohol advertisers, particularly because they can target messages and foster connections with consumers (Jernigan and Rushman 2014). This approach is of particular concern because it can easily reach adolescents and young adults under the legal drinking age. Software is available that would allow alcohol brands to ask for age verification before a user can become a follower of the brand’s account and interact with the brand. Such software typically requires the user to enter a birth date indicating that the user is over the legal age to purchase alcohol. However, a recent inquiry into alcohol brands found that none used any external age verification (Jernigan and Rushman 2014).

## Influence of Social Media on Young People

The influence of social media alcohol displays on young people can best be determined using theories that illuminate mechanisms of behavior change. Two classic theories in this respect are Social Learning Theory, which supports the importance of peer influence on behavior, and the Media Practice Model, which supports the role of media choices as influences on intentions and behaviors. A newer conceptual approach, the Facebook Influence Model, ties together many previous constructs from health behavior theory to understand how sites such as Facebook may be associated with these underlying constructs.

### Social Media Influence: Health Behavior and Media Theory Considerations

Social Learning Theory posits that adolescents learn both by direct experience and by observation (Bandura 1977, 1986). Previous work has indicated that observation of peers is a major source of influence on adolescent health attitudes, intentions, and behaviors (Keefe 1994; Wood et al. 2004). In particular, early alcohol initiation is determined at least in part by alcohol use by adolescents’ friends as well as by social network characteristics (Ellickson and Hays 1991; Mundt 2011). Thus, according to Social Learning Theory, observation of peers influences alcohol use intentions and behaviors. In today’s world, this observation may occur both online and offline.

The Media Practice Model states that adolescents choose and interact with media based on who they are, or who they want to be, in that moment (Brown 2000). This model suggests that media users explore information or display content based on experiences or behaviors they are considering, which may lead to reinforcement or advancement of these ideas. Thus, an adolescent who is considering initiating alcohol consumption may choose to watch a movie depicting drinking at a party, which in turn may influence him or her to attend such a party in the future.

Exposure to alcohol or tobacco in traditional media (e.g., movies, television) has been associated with adolescent substance use (Dalton et al. 2003, 2009; Gidwani et al. 2002; Titus-Ernstoff et al. 2008). Social media can combine traditional media exposure to alcohol-related content with peer interactivity (e.g., peer endorsement of specific behaviors), resulting in a potentially even more powerful influence on drinking behavior. For example, adolescents’ social media ties within and across networks provide many potential paths of influence. These paths may allow the spread of alcohol-related content or promote alcohol behaviors within a network as well as across networks (Mundt 2011). The potential impact of such messages has been demonstrated repeatedly. Thus, adolescents who view alcohol references on their peers’ Facebook profiles find these to be believable and influential sources of information (Moreno et al. 2009*a*). Furthermore, adolescents who perceive alcohol use as normative based on Facebook profiles are more likely to report interest in initiating alcohol use (Litt and Stock 2011). Consequently, social media represent a widespread, readily available, and consistently accessed source of information for today’s adolescents and young adults and combine the power of interpersonal persuasion with the reach of mass media. Fogg (2008, p. 23) described “mass interpersonal persuasion” as “the most significant advance in persuasion since radio was invented in the 1890s.”

## The Facebook Influence Model

A new evaluation of existing health behavior theory models is needed to understand the role of technologies such as social media (Collins et al. 2011). To address this issue, a recent study (Moreno et al. 2013*b*) sought to determine young people’s perceptions of which aspects of Facebook are influential. The mixed-methods study applied concept-mapping methodology, a validated five-step method to visually represent complex topics (Trochim et al. 1994). This approach allows the conceptual framework to be built from data based entirely on the views of key stakeholders and resulting in a concept map that visually represents key concepts and their interrelationships.

**Figure 2:**
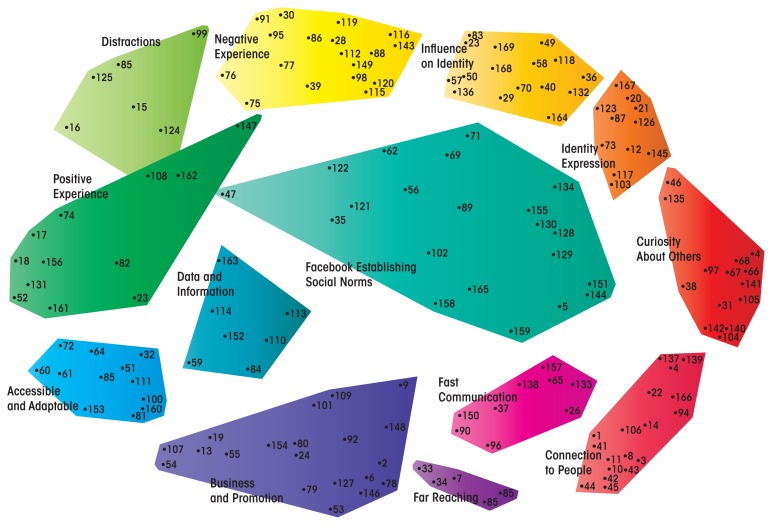
The Facebook Influence Model is a concept map created through Concept Mapping methodology. Each dot represents a single concept described by multiple participants in response to the question, “What makes Facebook influential?” After participants generated a list of concepts through a brainstorming process, they sorted these concepts into groups and ranked their importance. The map was then generated using Concept Mapping software employing a hierarchical cluster analysis to create a visual representation of the ideas arranged into clusters. Items that were similarly categorized by participants appear closer together on the map compared with items not categorized together. SOURCE: Moreno, M.A.; Kota, R.; Schoohs, S.; and Whitehill, J.M. The Facebook Influence Model: A concept mapping approach. *Cyberpsychology, Behavior and Social Networking* 16:504–511, 2013. PMID: 23621717

The resulting Facebook Influence Model includes 13 clusters representing specific aspects of Facebook, such as “influence on identity,” “connection to people,” and “social norms” (Moreno et al. 2013*b*) (see figure 2). The impact of these 13 clusters can be determined when classifying them into the following 4 categories or concepts characterizing the role of Facebook (see table):

- *Connection:* Facebook provides and enhances peer communication, networking, and connection.
- *Comparison:* Comparison with peers has long been a part of adolescence. Facebook allows this comparison using tangible information, such as photos and stated behaviors, as well as the ability to note peer comments on this information.
- *Identification:* Facebook allows the profile owner to develop an online identity through his or her profile. Profile owners can then reflect and revise that identity via feedback from peers’ comments and “likes,” or by personal perusal through the Facebook “timeline.” The ability to develop one’s identity in real time provides a unique multimedia view of the self.
- *Immersive experience:* Facebook has been described as a Web site that provides positive, negative, tool-based, and distracting features toward an immersive and powerful experience for users.

Moreno and colleagues (2013*b*) concluded that although Facebook provides a novel lens through which to consider factors that impact behavior, its influence can best be considered in the context of robust behavioral theory. Thus, each of the four concepts or cluster groups can best be considered alongside the framework of previous supporting work as synergistic with or an expansion of previous theory. For example, the “identification” concept describes the clusters that reflect how users explore and reflect on their identity using Facebook. As mentioned earlier, the Media Practice Model posits that users choose and interact with media based on how they perceive their identity at that time or what they would like their identity to be (Brown 2000). Facebook allows users to develop an online identity through their profile, which they can then reflect on and revise as described above. As a result, young people can develop an online identity in real time, based on a vision of who they want to be as well as exposure to other media content and peer feedback.

Further exploration of these 13 constructs and 4 concepts will provide a comprehensive base for theoretical consideration to inform future work and the potential for intervention development using Facebook.

## Social Media and Alcohol-Related Interventions

Despite the broad reach of social media, the literature to date is scant on interventions using social media to reduce harmful alcohol consumption. Consideration of previous work may help suggest future directions for social media–based interventions.

### Facebook

Based on previous work that identified links between displayed alcohol references on Facebook and self-reported alcohol behaviors (Moreno et al. 2011), one possible avenue for intervention could involve identifying individuals who may be at risk for alcohol-related problems based on the social media content they post. Screening these displays may represent innovative means to identify at-risk individuals and prompt them to undergo further screening and intervention. Studies have investigated young people’s willingness to engage in such interventions (Moreno et al. 2012*a*) as well as communication strategies for those who approach young people who display online content that is worrisome (Whitehill et al. 2013). Important issues to consider for interventions targeting specific individuals include how to identify those individuals given variation in privacy settings and the fact that the identity of social media users is not always known.

Facebook also provides opportunities to link user-generated content to triggered Facebook advertisements. As described in the Facebook Influence Model, this medium had a significant influence on “identity development,” and interventions could build upon this source of influence (Moreno 2013*b*). For example, researchers could consider linking Facebook advertisements to a user’s displayed alcohol content. These advertisements could provide messages for a user to consider when deciding whether to display alcohol content as part of an online or offline identity. Such advertisements could be triggered by certain keywords (e.g., terms related to “intoxication”) in Facebook posts and could include such messages as “Do you really want being drunk to be part of your identity?”

### Twitter

The relatively large volume of public content on Twitter suggests that it may be possible to implement an automated search system that would identify tweets indicating risk of alcohol-related problems and respond with a link to resources or services. However, an ongoing study to deter mine the feasibility of responding to tweets mentioning the words such as “drink,” “drunk,” or “drunk drive” found that unless the sender of the response tweet is already a follower of (or followed by) the targeted user, any tweets with a link are blocked by Twitter’s spam filter (Whitehill et al. 2014). Thus, the possibility of public health agencies conducting such efforts may be limited. Additional efforts to understand and test the ability to use various social media sites for automated two-way communication to reduce alcohol risk are needed.

### Social Media Advertisements

Another possible approach is to use social media for social marketing. In this way, social media could be used similarly to how traditional media outlets have promoted responsible alcohol use and increased awareness of alcohol-related harm. Advertisements could be pegged to the same keywords used by alcohol beverage advertising, with the goal of reaching the same target audiences and providing educational messages or links to online interventions.

### Mobile Devices

Other potential approaches to interventions may be based on the widespread use of social media sites from mobile devices, raising the potential that social media could be used to reach individuals in real time in the settings where drinking occurs. One pilot study of alcohol-using college students indicated that 42 percent used Facebook or Twitter during a drinking festival (Whitehill et al. 2012). Both Facebook and Twitter allow users to use the GPS feature of their phone to check in at their current location, and some specialized social-networking sites such as FourSquare allow users to locate friends nearby. It may be possible to use social media–based advertising and the location-based features of mobile phones to promote alternatives to drinking, safe transportation, free condoms, and other services to reduce the harms associated with alcohol consumption. Before such interventions could be developed, however, formative work is needed in this area to better understand the behavior of young people as it relates to their mobile social network use during the course of a drinking episode.

## Future Research Directions

Adolescents and young adults are particularly vulnerable to the effects of social media because they are at once early adopters and nearly ubiquitous users, as well as highly susceptible to peer influences (Ellison et al. 2007; Lenhart and Madden 2007; Lenhart et al. 2005, 2010). However, the field of social media research in this population is still in its infancy, and further work is needed in several arenas.

First, studies should expand and deepen observational research on social media sites. Past studies have described the content and timing of posts on sites such as MySpace, Facebook, and Twitter (Hinduja and Patchin 2008; Moreno et al. 2009*b*, 2010*a*; West et al. 2012). According to McCreanor and colleagues (2013, p. 119), “currently research is preliminary and descriptive, and we need innovative methods and detailed in-depth studies to gain greater understanding of young people’s mediated drinking cultures and commercial alcohol promotion.” Thus, studies have not yet fully harnessed the social aspects of social media by studying interactions between peers, distribution of content through a social network, or interactions between adolescents and adults. These types of studies would help deepen our understanding of how alcohol content is distributed and shared through networks and potentially identify intervention partners who have access to and are willing to confront adolescents and young adults regarding displayed references to alcohol.

Second, researchers should further explore the interactive nature of social media sites that provides new opportunities for interventions. Such interventions must be developed with an understanding of the privacy settings within each network. Only individuals who are able to view the content and are comfortable communicating about it would be able to conduct such interventions. Understanding to what extent parents, teachers, college resident advisors, and other influential adults are privy to young people’s displays of alcohol content on social media is an area for future inquiry. For example, if parents see a reference to problem alcohol use on their child’s Facebook profile, that reference may indicate that the child actually engages in problem drinking. Parent–child communication prompted by that social media reference could have an important impact. Preliminary work has explored communication strategies for these encounters and potential intervention opportunities (Moreno et al. 2012*a*; Park and Calamaro 2013; Whitehill et al. 2013). However, additional work is needed to understand how this knowledge can be translated into clinical practice or educational interventions appropriate to different settings, such as schools, clinics, or universities (George et al. 2013).

Third, research should explore the extent to which young people are exposed to advertising from alcohol manufacturers across social media sites. Regulations or new technology-based methods to avoid displaying such content to underage individuals may be possible and warranted. The same social-marketing approaches that may be used to promote alcohol on social media also can potentially be harnessed to promote abstention before age 21 and responsible use thereafter. In these ways, an improved understanding of the new landscape of social media could be used to reduce the negative consequences of alcohol use among youth.

In all future studies involving social media sites, attention to two factors will be critical. First, researchers must pay attention to privacy settings on the sites and the users’ expectations for protection of their confidentiality. Previous work has illustrated that older adolescents are willing to interact with others regarding their displayed health risk behaviors on social media sites (Moreno et al. 2012*b*); however, differences may exist between social media sites with respect to users’ expectations for privacy. Second, it is crucial to approach social media sites with the same ethical and regulatory rigor that is expected in offline studies involving human subjects. To this end, several guidelines have been published that identify best practices in study design and working with institutional research boards (Moreno et al. 2009, 2013*a*; Sixsmith and Murray 2001; Zimmer 2010).

## Conclusion

Social media have a broad reach into the lives of many young people and therefore have the potential to strongly influence their decisions. The growing body of literature on social media and alcohol suggests that researchers can consider the role of social media in alcohol consumption in two ways. First, social media can serve as a source of information about the behavior of the individual user, as illustrated by studies that link online content to offline behavior (Moreno et al. 2011) or demonstrate links between online and offline alcohol consumption patterns (West at al. 2012). Second, social media can be a source of influence on behavior according to such behavioral models as Social Learning Theory (Bandura 1986), the Media Practice Model (Brown 2000), and new theoretical frameworks such as the Facebook Influence Model (Moreno et al. 2013*b*). The influence of alcohol advertising in social media is not yet fully understood. Future work is needed to broaden our understanding of alcohol content across social media sites and over time in an adolescent’s development. Preliminary studies have begun to investigate possibilities for interventions using social media (Moreno et al. 2012*a*; Park and Calamaro 2013; Whitehill et al. 2013). Additional studies should integrate observational data, health behavior theory, and intervention possibilities to fully harness the tools social media may offer in the public health arena.

## Figures and Tables

**Table:** Characteristics of the Different Clusters in the Facebook Influence Model

Domain	Cluster Label	Example Items Within Cluster
Connection	Connection to people	–Allows people to constantly stay updated with other’s lives –Way to get to know acquaintances almost instantly –Keep in touch with people you would not call or text
	Far reaching	–Ability to reach many people with one Web site –Can reach anyone, young and old, rich and poor –Bonding across cultures and distances
	Fast communication	–Feel connected and in the loop constantly –Puts everyone you know and what they are doing in one place –Updates on people’s lives faster than with a cell phone
	Business and promotion	–Ability to plan influential events such as protests or sit-ins –Statuses provide a way to blog instantly about events or political topics –Every company uses it to promote business or provide deals
	Accessible and adaptable	–Largest network in human history –Easy to use and navigate –Widely known and talked about
	Data and information	–Huge database of information –Compiled data from millions of individuals –News feature

Identification	Identity expression	–Freedom to express things and let them be heard –Present the best side of yourself –Show off accomplishments to everyone you are friends with on Facebook, not just close friends
	Influence on identity	–Provides others with pictures that can influence perceptions –Display aspects of yourself that you would not share in offline life (sexuality, substance use) –Wonder if you should be doing what you see everyone doing in pictures

Comparison	Curiosity about others	–Can know what people are up to without asking them about it and without them knowing you know –Creep culture/stalking –See who associates with whom with pictures and comments
	Facebook establishing social norms	–Reinforces beliefs or opinions by seeing that others hold same beliefs or opinions –Can see what is popular by observation –Can follow norms

Facebook as an experience	Distractions	–Procrastination –Addictive –Huge distraction
	Positive experiences	–Facebook is referenced in daily life –Provides entertainment at any time –Status updates can promote a good mood
	Negative experiences	–Changes the nature of communication from face to face to screen to screen –People willing to sacrifice privacy –Inspires competition in people

SOURCE: Moreno, M.A.; Kota, R.; Schoohs, S.; and Whitehill, J.M.; The Facebook Influence Model: A concept mapping approach. *Cyberpsycholology, Behavior, and Social Networking* 16(7):504–511, 2013. PMID: 23621717
